# Tackling disrespect in health care: The relevance of socio-relational equality

**DOI:** 10.1177/13558196231187961

**Published:** 2023-07-27

**Authors:** Vikki A Entwistle, Alan Cribb, Polly Mitchell

**Affiliations:** 1 Professor of Health Services Research and Philosophy, Health Services Research Unit and Department of Philosophy, 1019 University of Aberdeen, Aberdeen, UK; 2 Professor of Bioethics and Education, School of Education, Communication and Society, 121212 King’s College London, London, UK; 3 Research Fellow, School of Education, Communication and Society, 121212 King’s College London, London, UK

**Keywords:** respect, equality, social justice

## Abstract

Disrespect in health care often persists despite firm commitments to respectful service provision. This conceptual paper highlights how the ways in which respect and disrespect are characterised can have practical implications for how well disrespect can be tackled. We stress the need to focus explicitly on disrespect (not only respect) and propose that disrespect can usefully be understood as a failure to relate to people as equals. This characterisation is consonant with some accounts of respect but sometimes obscured by a focus on respecting people’s autonomy and dignity. Emphasising equality is consistent with connections patients draw between being (dis)respected and (in)equality. It readily accommodates microaggressions as forms of disrespect, helping to understand how and why experiences of disrespect may be unintentional and to explain why even small instances of disrespect are wrong. Our view of disrespect with an emphasis on equality strengthens the demand that health systems take disrespect seriously as a problem of social injustice and tackle it at institutional, not just individual levels. It suggests several strategies for practical action. Emphasising relational equality is not an easy or short-term fix for disrespect, but it signals a direction of travel towards an important improvement ambition.

## Introduction

Patients with complex needs following hip fracture or stroke felt “invisible” while hospital staff talked over and about them and took no notice of their concerns about when and to where they would be discharged. Some were moved at short notice early in the morning to a room that nurses described as a “holding pen”, then transferred late at night to another facility with little information about where they were going or for how long.^1^

Black and Asian women in the UK experience pervasive microaggressions during pregnancy and childbirth. Their concerns are often ignored or dismissed by healthcare professionals, sometimes due to racial stereotypes. They are much more likely to die in pregnancy or childbirth than white women.^2,3^

Many negative experiences of health care can be categorised, at least loosely, as disrespect. These include being talked down to, not taken seriously, unfairly judged and prejudicially discriminated against. Some occurrences of disrespect are palpably problematic. Others are subtler and perhaps ambiguous, but can nonetheless contribute to a range of problems and harms that include: deterring access to effective and timely health care; reducing service efficiency via premature termination of service use; undermining emotional and psychosocial wellbeing and contributing to the kinds of error that undermine patient safety.^4–10^ Disrespect may be both especially prevalent and especially harmful for groups that are routinely socially marginalised.^4,7,8^

Problems of disrespect persist beyond high profile condemnations of shocking examples of institutionalised disrespect, and despite health care leaders stressing commitment to respectful practices. Projects have documented and sought to address disrespect and abuse in particular clinical areas and institutions, including maternity services and long-term care.^11–13^ Sokol-Hessner et al.^9^ developed a framework for reporting and analysing incidents of non-physical harm experienced by patients and/or families, and an approach to sharing coworkers’ concerns about disrespectful and unsafe behaviours has been demonstrated in at least one medical centre in the USA.^13^ Beyond that, there seem to be few practical plans and initiatives to tackle disrespect in its broader manifestations and as a matter of routine policy for health care systems.

Disrespect is a multi-faceted and complex social problem, and it would be a mistake to think that there is a quick fix that health service leaders should just get on with and implement. In this paper, we seek to support efforts to tackle disrespect in health care by considering how such efforts can be hindered or helped by different ways of conceptualising respect and disrespect.^14^ We contribute as applied philosophers interested in the practical value of conceptual and ethical analysis, and we draw on our own experiences of health care encounters as well as the accounts of others and various literatures on respect. We propose an understanding of disrespect as failure to relate to all people as fundamentally equal as a practically important but neglected complement to (related) understandings of disrespect as failure to recognise and respond appropriately to human dignity. Considering disrespect as a failure in relational equality strengthens the case for tackling disrespect as a matter of justice and suggests a new strategic focus for action.

Before we make our case for an emphasis on equality, we outline some challenges to tackling disrespect in health care that are at least in part conceptual. We then briefly review philosophical accounts of respect and suggest that more explicit emphasis could be put on the idea of fundamental human equality that is implicit in some accounts of respect. We develop this idea by drawing on accounts of socio-relational equality and microaggressions. We then highlight the implications of a view of disrespect that emphasises socio-relational equality. For concision, we focus on health care systems and professionals showing disrespect for patients. However, most points apply to the linked and serious problems of disrespectful treatment of family members and health care staff.

## Conceptual and related challenges to tackling disrespect in health care

The terms ‘respect’ and ‘disrespect’ are widely used, but their meanings are often ambiguous: people may be said to respect and disrespect different kinds of things, in various ways, and for different reasons.^15^ Thus, when policy makers, health service leaders, improvement advocates and others talk about disrespect, they may have somewhat different concerns in mind. Talking at cross purposes or with different emphases can be a problem for coordinated action, but that is not the only conceptual challenge to our collective ability to tackle disrespect in health care.

Health care mission and value statements are replete with statements to the effect that patients and staff should be treated with respect. We agree that this is important to affirm. However, if disrespect is not addressed explicitly, there is a risk it is seen as a mere lack of something desirable rather than something that is wrong and seriously harmful.

Respect and disrespect as generally understood have both attitudinal and behavioural aspects. They can be expressed in diverse ways, both verbal and non-verbal, at interpersonal and broader social institutional levels, including via micro-communication (for example, single words or short phrases, expressive eye movements, and gestures or sighs that subtly show esteem or disparage people) and absence of attention. The signalling of respect and disrespect can be more and less intentional and what communicates respect and disrespect varies to some extent across social contexts. The attitudes of respect or disrespect that one person holds or believes they express to another will not always coincide with the other’s experience of being respected or disrespected.^15,16^ A patient can feel disrespected by a health professional who has no intention of disrespecting them.

Several features of disrespect make it difficult to recognise confidently and to measure, including that its manifestation can be micro, that it can occur in dynamic and unwitnessed interactions and that its interpretation is contestable. This can impede policy and health care improvement action because these tend to require clear and quantitative evidence of problems and progress. Uncertainty about interpretation and difficulties evidencing people’s intentions can also reduce confidence about tackling occurrences of disrespect: there are reasons for caution about calling people out for communication they may not have intended as disrespectful, especially if this has blaming and shaming effects.^17^

The micro-communicative form of many expressions of disrespect can also make them seem relatively insignificant. Taken in isolation, one small instance of disrespect seems unlikely to do much harm. This may lessen the perceived need to confront it.^17^

Efforts to speak up about the disrespect experienced by patients often go unheard, especially when those speaking also belong to otherwise marginalised groups, for example, by virtue of their sex, skin colour, age or disability. Marginalised people are particularly likely to be ignored, silenced, misinterpreted or discredited when they try to express concerns about their treatment.^17^ Even health care leaders who firmly intend to be respectful can struggle from their positions of privilege to comprehend and recognise some experiences as experiences of disrespect. If people’s claims of being disrespected are persistently not listened to, understood or treated as credible, they may stop trying to make them. Thus, disrespect can be repeated and amplified in feedback loops.

Impetus and scope to tackle disrespect may be further limited when respect is closely tied to concepts like patient-centred care and its near synonyms. This is ironic because these concepts were developed against tendencies to disrespect patients by reducing them to their disease or body parts or otherwise neglecting important aspects of their humanity. In practice, however, the scope and salience of respect in health care has often been attenuated within conceptions of patient-centredness. For example, a characterisation of patient-centred care as “Respectful of and responsive to individual patient preferences, needs and values, and ensuring that patient values guide all clinical decisions”^18^ ^p.6^ is often interpreted with an emphasis on treatment decisions and a focus on ensuring patients are informed and given a say about offered health care options. While information provision and involvement in treatment decision-making can be very important, respect for patients as choice-makers is somewhat narrower than respect for patients as people more generally. A similar point applies to the reduction of an ethical principle of respect for persons to one of respect for autonomy.^19^ Many troubling examples of disrespect occur when patients are not making, and may not be able to make, treatment decisions. Advocacy of respect for patients’ choices will not necessarily avoid, and may even exacerbate, other forms of disrespect.

For all these reasons, problems of disrespect are sometimes obscured and left unaddressed. Health care leaders who seek to tackle disrespect need an account that clearly highlights when and why it is wrong and harmful (including in its micro-manifestations) and that can otherwise help overcome the conceptual sources of inertia against tackling it.

## Philosophical accounts of respect

Philosophers distinguish between different forms of respect.^15^ Their debates are not fully settled, but a distinction between ‘appraisal respect’ and ‘recognition respect’^20^ is helpful for thinking about what kinds of disrespect are problematic for health care.

‘Appraisal respect’ is a positive assessment of the moral traits or other qualities of a specific person that relate to their character, on the basis of which we think more highly of them.^20^ We can have different degrees of appraisal respect, and for some but not all of someone’s characteristics.^15^ For example, we might have significant (appraisal) respect for a doctor on account of their commitment to improve patient safety but not on account of their being unfaithful to their spouse.

‘Recognition respect’, by contrast, involves having due regard for something, and awarding it a weight in our decision-making that is appropriate for the kind of thing that it is. We can show recognition respect to laws, to religious leaders, to the natural world and more, but recognition respect is perhaps most often used to describe the regard that is due to all human beings. The basic, but not simple, idea is that we should recognise and respond appropriately to the significant moral worth that all human beings possess as such.^15^ There are debates about whether this moral worth is best grounded in some common human characteristics, intrinsic human dignity and/or membership in the human community, but these need not trouble us here because, however they are settled, the appropriate (respectful) responses that this significant moral worth demands of us include that we give each person due acknowledgement, attention and value weighting in our attitudes and behaviour.^15,20^

Recognition respect does not depend on what we like or dislike about any specific people: it is a matter of correctly identifying and responding to a moral status that all people share. The basic idea of recognition respect is consistent with describing experiences of disrespect as dehumanising. Failures of recognition respect are also inappropriate responses to people’s fundamental *equality*. This is at least implicit in many accounts: our common humanity underpins our significant *and equal* moral worth. We suggest that emphasising this equality more explicitly may be useful for tackling disrespect.

### Disrespect and equality

In health services research and policy, equality is typically considered with a focus on patterns of distribution and questions of who has what share of important goods, including health and access to health care. Concerns are raised when some social groups have, on average, more or less of these goods than other groups. In contrast to this distributive approach, some philosophers adopt a social or relational approach, considering equality as first and foremost about relationships between people.^21,22^ Socio-relational accounts of equality vary in detail but share a focus on questions of whether and how people relate to each other as equals, and how people should interact in a society of equals. Their answers refer to a cluster of concepts in which recognition and respect feature prominently along with reciprocity, solidarity, non-domination and avoidance of oppressive uses of power.^21^ Socio-relational egalitarians generally think people should interact in ways that reflect and reinforce a commitment to something like recognition respect, with behaviours that are appropriately responsive to the moral worth and equal entitlement to recognition that are shared by all human beings. They do not deny the importance of distributive patterns, but their views about which patterns matter and why reflect their thinking about how people should relate to each other.

Connecting these ideas, we propose to consider disrespect as failure to recognise and respond appropriately to someone’s fundamental human equality. We can also say that disrespect involves relating to other people as inferior beings.

### Disrespect and microaggressions

This way of understanding disrespect can readily accommodate microaggressions as significant examples of disrespect. Microaggressions are increasingly recognised as forms of disrespect that are widespread and can cause harm in health care contexts.^5,8,17,23^ Regina Rini defines a microaggression as “a small act of insult or indignity, relating to a person’s membership in a socially oppressed group, which seems minor on its own but plays a part in significant systemic harm”^17, p. 2^ because it results from, and contributes to, socially unjust conditions in which some groups are systematically not treated as fundamentally equal.

On Rini’s account, microaggressions are experienced ambiguously because the intentions of those who commit them may be unclear or difficult to evidence. For those on the receiving end, the uncertainty of the experience brings a cognitive and emotional burden, a wondering and worrying about what was intended and what the implications are. Long, complex histories of oppressive social discrimination may lead people in privileged groups and positions inadvertently to microaggress despite having no intention to be disrespectful. They might, for example, be unaware of the origins and problematic, ‘put-down’ implications, perhaps sexist, racist or ableist connotations, of a word they use. Or they might recently have become aware of these connotations but still accidentally use the word out of habit.^17^

Members of minoritised groups are often subject to many microaggressions as well as more blatant instances of disrespect. All the small instances amount to bad treatment that cannot appropriately be ignored; Rini used the analogy of being able to deal with one bee and its sting but not an attack from a whole swarm.^17^

## Disrespect with an emphasis on socio-relational equality: Implications for health care

In this final section, we briefly explain the relevance of an equality-focused view of disrespect for health care contexts, then illustrate its potential both to strengthen the arguments for tackling disrespect (as a matter for justice) and to offer practical strategies for action.

Socio-relational ideas about equality were developed primarily to think about the equality of citizens. Their relevance to health care might be questioned because relationships between health professionals and patients have specialised purposes and are inevitably asymmetric in important respects. Health professionals have higher levels of expertise and authority in some health-related domains, and they have legally and professionally regulated responsibilities towards patients, who are sometimes deeply dependent on them for care. We agree that these are important considerations, but they do not constitute grounds for rejecting socio-relational ideas about equality as relevant for identifying and tackling disrespect in health care. The central claim of socio-relational accounts of equality, that people should be treated as moral equals on the basis of their shared humanity, serves to identify and highlight the inappropriately differential treatments that tend to arise from poor responses to human variation and to social inequality in other senses.

Acknowledgement of different forms and degrees of expertise, knowledge, and (to some extent) institutional and social responsibility and status is compatible with the idea that, fundamentally, all people have equal moral worth. Treating people as equals does not mean treating everyone the same in every way or discounting individual capabilities and merits, but socio-relational egalitarians will look critically at how various differences between people are handled in interactions. One core tenet, which underpins much human rights legislation, is that differences in characteristics such as sex, race or ethnicity and dis/ability do not render people less than fully human, and it is not warranted to discriminate against people, that is, to relate to people as if they are inferior, on these grounds. This idea can be extended to cover grounds of stigmatised health conditions, health-related behaviours and patienthood.

Several observations from health care practice and health services research support adopting a view of disrespect that emphasises socio-relational equality. First, when health professionals treat patients or colleagues with such serious disdain that they humiliate them, they are widely agreed to behave disrespectfully (and wrongly). Health professionals’ role responsibilities and often high levels of biomedical knowledge and skill neither necessitate nor justify arrogant behaviour or oppressive abuses of power. Second, patients consider experiences of equality between health professionals and themselves relevant, not only to questions of how good health care is but also to ideas of respect. Many of the expressions that patients use to describe poor health care reflect experiences of having been related to as less than equally human. They talk, for example, of being treated like ‘an animal’, ‘vermin’ or ‘dirt’, as well as being made to feel ‘small’, ‘invisible’ or otherwise diminished in significance. Being treated as an equal is a key theme when patients describe respectful health care.^8,17,23–25^

### Strengthening the case for tackling disrespect: A matter of justice

A view of disrespect as failure to recognise and respond appropriately to someone’s fundamental human equality helps explain why disrespect is wrong. It accommodates an understanding of microaggressions that in turn explains why the wrong of even apparently small instances of disrespect should be taken seriously even if direct evidence of harmful consequences is lacking. Microaggressions are rooted in, and serve to reinforce, the widespread treatment of some groups as less than equal. Once we appreciate this, neither the wrong nor the harm of microaggression can be considered insignificant:^5,17^

[I]n cases of background injustice… unequal treatment of others, which might otherwise be considered merely morally dubious, can become part of an ongoing societal practice of injustice.^21, p. 14^

If health care systems’ rules of engagement, organisational messaging or the more directly communicative behaviours of staff, reflect the discriminatory norms operating in broader societies, they also contribute to the ongoing normalisation of oppression.^17,26^ To the extent that disrespect, including in its micro-manifestations, is not proactively tackled, our view suggests that health care systems are not just acquiescing to but reinforcing social injustices. This is also the case when institutionalised disrespect of people with some health conditions or health-related behaviours creates and sustains an underclass of patients.

### Practical strategies for action

Strategically, emphasising disrespect as a failure to enact socio-relational equality orients attention towards relationships and the hierarchies between people that can be reflected in and generated by health care systems, organisational processes and staff behaviours. It demands careful vigilance against treating some people as having inferior moral status. Table 1 illustrates some practical applications of this way of thinking about disrespect.

**Table 1.** Examples of disrespect understood with an emphasis on socio-relational equality.

Situation and event	Why is this disrespectful?	How could these kinds of disrespect be avoided?
A patient wearing only a short hospital gown sits, as instructed, on a plastic chair outside a changing cubicle in an otherwise empty corridor. She is waiting to be taken to a diagnostic imaging room. A uniformed health professional enters the corridor and starts to change the notices on each of the cubicle doors. They ignore the patient.	The combination of hospital gown and corridor waiting location put the patient in an inferior position and render her vulnerable to further disrespect as they prevent her from presenting to others on usual equal (fully clothed) terms. Although the health professional in the scenario may not be responsible for clinical aspects of the patient’s care, they are in the kind of proximity in which acknowledgement of another human being is usually socially warranted. By ignoring her, they could be seen to suggest that she does not merit this usual human attention. Her (and our) wondering and worrying about this suggestion would be heightened if she might be taken for a member of a minoritised group, for example, if she had a darker skin colour, visible disability or ambiguous secondary sex characteristics or if she was obese. If the health professional is a man, sexism might also be a concern. The fact that they are a health professional and she a patient, and a patient facing the emotional and other challenges that diagnostic imaging would raise for most human beings, exacerbates the implication of them not treating her as worthy of a conventional basic acknowledgement.	At an institutional or systemic level, a redesign of hospital spaces, gowns and processes might reduce patients’ inferior positioning and general vulnerability to disrespect. In terms of professional behaviour, in the given context, a simple greeting ‘hello’ or even a perceptible nod of acknowledgement of the patient’s human presence on the part of the health professional could have made an important difference. A smile and a brief but friendly comment would have gone further to signal recognition of socio-relational equality, particularly if the comment recognised something of the challenge of the patient’s position, for example, by expressing some hope or justified reassurance that she would not be kept waiting there for long. If the staff member is themself feeling disrespected by colleagues, managers or the broader system or society in which they work, this may make it harder for them to communicate respectfully with the patient, and needs to be tackled as part of the problem.
A nurse conducting a routine consultation berates a person with diabetes for putting on more weight and not improving her diet or increasing her exercise levels. The nurse repeats what they have told this person several times before about needing her to understand that she is much more likely to suffer heart attack, stroke, diabetes-related blindness and amputations if she does not get her blood glucose and blood pressure levels down. The nurse stresses a need to try harder to achieve at least a small weight reduction by the time of the next appointment.	While it is important for health professionals to explain the rationales and evidence behind their recommendations, the nurse’s assumption that the person does not understand and has not tried, and their lack of attention to the patient’s perspective, reflects a failure to recognise and respond to the patient as a moral agent. The patient may well have understood the information the nurse has given her and tried to do something about her diet but be facing significant practical challenges in her social circumstances. To some extent, the nurse’s rather blinkered focus on the patient’s weight, blood glucose and blood pressure might reflect professional and health system emphases that tend to crowd out both broader and patient-specific considerations of what matters for health and wellbeing and more generally tend to dehumanise. However, it is also possible that the nurse is actively seeking to maintain their superiority in the relationship; health professionals sometimes communicate strategically to preserve a hierarchy not only of expertise but also control over patients, and a view of patients as dependent on them.^30^	At an institutional or systemic level, attention should be paid to the ways in which biomedically driven policies, protocols and standards can constrain the broader humanity in health professionals’ interactions with patients. Supportive peer ‘calling in’ of unnecessarily hierarchical behaviours might be encouraged in respectful professional development and mentoring programs (Rini’s discussion of proleptic blame is useful here).^15^ For individual health professionals, a non-judgemental inquiry into the person’s own perspectives on managing their diabetes would signal some recognition of their moral agency and create an opportunity to demonstrate sufficient empathy to signal some sense of human equality. A variety of comments and questions could serve to indicate that the nurse appreciates that the person, like other humans including the nurse, has an interest in their own life and personal priorities for how it goes. At both levels, an orientation to support people to live well with diabetes is more readily compatible with a commitment to avoid disrespect than an orientation to motivate them to work towards narrower biomedical goals.^31^
A patient waiting on a trolley in a hospital corridor tells a passing health professional that he is in pain and asks if they know when he might be attended to. The health professional replies as they walk past, saying that this is not a private hospital, they are currently short staffed, and there are some higher priority patients who must be seen first.	The health professional, overstretched as they are, might have simply been trying to explain to a patient who was due to be seen by staff from another team why he had not yet been attended to. Several features of the wording and delivery of their reply could, however, be interpreted as disrespectful. For example: they ignored the report of pain (widely recognised as a human distress signal); their mention of private hospitals and higher priority patients (without any indication of the basis of prioritisation) could suggest this patient is seen as of lesser value than others and is seen as selfish or demanding; their brevity could suggest an impatient dismissal of the person and their concerns; and the lack of empathic connection in their communication could be taken as a failure to relate to the patient on an equally human level. The disrespect within the encounter would arguably be worse if the patient has characteristics that tend to be discriminated against and the health professional has characteristics associated with social privilege. Persistent inaction against gross disparities in access to good quality health care can communicate disrespect on the part of health care systems and their improvement teams. They may constitute an acquiescence in injustice.	The avoidance of disrespectful (as well as otherwise inadequate) health care is difficult without sufficient qualified staff and facilities to support timely attention to patients’ needs. At governmental, systemic and institutional levels, leaders in positions to influence working conditions must take significant responsibility for this kind of situation. Health professionals who recognise and respond to patients as fundamentally their equals are likely to be disappointed when the service, they contribute to falls short of the standards of response to human need that they themselves would want to be treated by. At the level of the individual health professional, some acknowledgement of their disappointment can signal to patients that the health professional does not see them as inferior or as not deserving of the standards they would expect for themselves. A simple ‘sorry’ or expression of regret about service shortfalls can indicate that no disrespect is intended on their part. A brief acknowledgement of the difficulty of the patient’s situation could also help by signalling some connection at a human level.

When characterised as a failure to recognise and respond to people’s fundamental equality and understood in the context of histories of widespread discrimination, disrespect can also clearly be seen as a product and feature of complex social and health care systems, not merely one of individuals’ attitudes and behaviours. The treatment of (some) patients as inferior has arisen and continues to arise because some prejudicial hierarchies are deeply socially ingrained. An emphasis on socio-relational equality supports the case for tackling disrespect as a matter of policy and with attention to institutional systems as well as individual level attitudes and actions.

An emphasis on inequality can helpfully highlight how disrespect lands on some people more than others. In conjunction with Rini’s account of microaggressions, it shows why disrespect cannot simply be fixed by everyone intending to be respectful. Rini usefully proposes ‘proleptic blame’, that is, privately ‘calling in’ potentially inadvertent instances of microaggression with supportive intent that assumes people want to do better, to support people to drop habits and practices shaped by prejudicial social norms and structures without punitively blaming and shaming them.^17^

It is harder to avoid reinforcing relations of superiority/inferiority between different patients when access to health care generally or to particular and relevant services depends on resources of various kinds which some people lack. Health service resource constraints, especially problems of under-staffing, also impact health professionals’ ability to avoid disrespect. It may be possible for staff to be respectful in difficult working conditions but, realistically, overstretched and exhausted people are more likely to make relational, moral mistakes as well as other professional mistakes, and staff who are disrespected themselves face further challenges. These considerations add weight to the case for considering disrespect as a problem that needs to be tackled at all levels, including in policy and funding decisions.

In principle, our proposed emphasis on equality suggests that one key test of whether health care avoids disrespect is whether it effectively signals to people that they are recognised and responded to appropriately in their equal humanity. Monitoring and, where necessary, improving performance on this test will require careful attention to people’s subjective experiences. This is not simple or completely unproblematic, but service providers can use various strategies to understand when and why they may be falling short of ensuring people are related to as equals, including the development and evaluation of questionnaires that ask patients and family members whether they were treated as equals.^27^

It will be particularly important to monitor and learn from the experiences of socially minoritised groups. Not only are they more likely to experience disrespect; they may be particularly able to identify when and how inferiority is signalled and received. Diversity within groups working to tackle disrespect can also help ensure they have sufficient breadth of perspective and insight to recognise the socially hierarchical structures and unjustifiably discriminatory practices that reflect and generate relational inequality and need corrective attention.

As sensitivity to potentially unfairly discriminatory practices develops, analyses such as those reported in recent studies of the disrespectful language used in medical records, can help identify relational shortfalls and make specific behavioural recommendations for improvement.^28,29^ We suggest similar attention should be paid to the assumptions about patients that are built into service design and reflected in staff responses when people do not fit these assumptions, for example, when equipment does not cater for larger body sizes.

Another important focus will be on any differential relational treatment that is a response to personal characteristics. As noted above, relating to people as equals does not mean behaving in the same way with everyone; it can require responsive variations. For example, when working with people with cognitive or communicative impairments, language differences or lower literacy that tend to limit their participation in common forms of human interaction, health services and staff may need to adapt their usual forms of communication to avoid disrespect. The view of disrespect that we are advancing encourages careful attention to how such adaptations are made and what they signal about people’s relative status. Attention to the perspectives of diverse patients and their close supporters is likely important here to avoid falling into traps of condescension. Approaches to attending to such perspectives will also need to be considered critically.

People’s assessments of whether they have been treated as equals may depend both on their direct experiences of interaction with health care providers and on how their experiences compare with other patients' experiences. The latter, indirect or comparative, assessment of relationships explains why distributive patterns of patients’ experiences, outcomes and evaluations of health care can sometimes help identify where work to tackle disrespect may be particularly needed (Table 2).

**Table 2.** Summary of key points.

Various conceptual issues and associated challenges can hinder the tackling of disrespect:
• Talking positively about respect without explicitly committing to tackle disrespect tends to downplay the wrong of disrespect.
• If respect is thought of primarily as a matter of respecting patients’ choices or autonomy, many experiences of disrespect will be overlooked.
• Health care improvement initiatives tend to focus on what can be measured. It can be difficult to measure respect and disrespect in practice because:
○ Respect and disrespect are variously understood,
○ Respect and disrespect can be variously expressed, including in micro-communication,
○ Conventions for communicating respect and disrespect vary across cultures,
○ One person’s intention to respect another does not always ensure the other’s experience of being respected.
○ It can be difficult to attribute substantial harm to any particular instance of disrespect. This can seem to reduce the importance of tackling disrespect.
• It can be difficult to attribute substantial harm to any particular instance of disrespect. This can seem to reduce the importance of tackling disrespect.
There is scope to refresh and enhance ways of thinking about respect and disrespect:
• Philosophical considerations of recognition respect stress the importance of recognising and responding appropriately to people’s human dignity, humanity and moral worth.
• The idea that all humans possess these equally is sometimes mentioned but perhaps under-emphasised in considerations of disrespect and efforts to tackle disrespect in health care.
• An emphasis on socio-relational equality, that is, recognising and relating to people as equals, can help illuminate instances of disrespect and their moral significance and accommodate microaggressions as forms of disrespect.
Our proposed view of disrespect, which emphasises people’s fundamental equality:
• Is consistent with patients’ linking of respect and equality,
• Supports recognition of instances of disrespect that might go undetected or look more ambiguous and less problematic on other accounts,
• Supports those trying to make a case to invest in tackling disrespect by explaining that and why it is required as a matter of social justice,
• Offers practical strategic direction for action – extending the repertoire of strategies to tackle disrespect.

## Conclusion

A view of disrespect as failure to recognise and respond appropriately to someone’s fundamental equality of human worth helps to emphasise that disrespect is wrong irrespective of any additional harms it contributes to. A linked understanding of microaggressions as ambiguous experiences that arise from and perpetuate systems of unjust social discrimination supports the case for tackling disrespect as a matter of social justice.

An emphasis on disrespect as a failure of socio-relational equality suggests a strategic focus on relationships and efforts to identify and remediate the ways that health care systems and those who work within them create or reinforce social hierarchies in which (some) patients or groups are positioned and related to as less than equal. Putting an emphasis on socio-relational equality is not an easy or short-term fix for disrespect, but we suggest it is a direction of travel towards an important improvement ambition.
